# The presence of coexisting sleep-disordered breathing among women with hypertensive disorders of pregnancy does not worsen perinatal outcome

**DOI:** 10.1371/journal.pone.0229568

**Published:** 2020-02-26

**Authors:** Danielle L. Wilson, Mark E. Howard, Alison M. Fung, Fergal J. O’Donoghue, Maree Barnes, Martha Lappas, Susan P. Walker

**Affiliations:** 1 Institute for Breathing and Sleep, Austin Health, Heidelberg, Victoria, Australia; 2 Mercy Perinatal, Mercy Hospital for Women, Heidelberg, Victoria, Australia; 3 Department of Medicine, University of Melbourne, Parkville, Victoria, Australia; 4 Department of Obstetrics and Gynaecology, University of Melbourne, Parkville, Victoria, Australia; University of Mississippi Medical Center, UNITED STATES

## Abstract

**Objective:**

To determine whether the presence of co-existing sleep-disordered breathing (SDB) is associated with worse perinatal outcomes among women diagnosed with a hypertensive disorder of pregnancy (HDP), compared with normotensive controls.

**Study design:**

Women diagnosed with HDP (gestational hypertension or preeclampsia) and BMI- and gestation-matched controls underwent polysomnography in late pregnancy to determine if they had coexisting SDB. Fetal heart rate (FHR) monitoring accompanied the sleep study, and third trimester fetal growth velocity was assessed using ultrasound. Cord blood was taken at delivery to measure key regulators of fetal growth.

**Results:**

SDB was diagnosed in 52.5% of the HDP group (n = 40) and 38.1% of the control group (n = 42); p = .19. FHR decelerations were commonly observed during sleep, but the presence of SDB did not increase this risk in either the HDP or control group (HDP group—SDB = 35.3% vs. No SDB = 40.0%, p = 1.0; control group—SDB = 41.7% vs. No SDB = 25.0%, p = .44), nor did SDB affect the total number of decelerations overnight (HDP group—SDB = 2.7 ± 1.0 vs. No SDB = 2.8 ± 2.1, p = .94; control group—SDB = 2.0 ± 0.8 vs. No SDB = 2.0 ± 0.7, p = 1.0). Fetal growth restriction was the strongest predictor of fetal heart rate events during sleep (aOR 5.31 (95% CI 1.26–22.26), p = .02). The presence of SDB also did not adversely affect fetal growth; in fact among women with HDP, SDB was associated with significantly larger customised birthweight centiles (43.2% ± 38.3 vs. 16.2% ± 27.0, p = .015) and fewer growth restricted babies at birth (30% vs. 68.4%, p = .026) compared to HDP women without SDB. There was no impact of SDB on measures of fetal growth for the control group. Cord blood measures of fetal growth did not show any adverse effect among women with SDB, either in the HDP or control group.

**Conclusion:**

We did not find that the presence of mild SDB worsened fetal acute or longitudinal outcomes, either among women with HDP or BMI-matched normotensive controls. Unexpectedly, we found the presence of SDB conferred a better prognosis in HDP in terms of fetal growth. The fetus has considerable adaptive capacity to withstand in utero hypoxia, which may explain our mostly negative findings. In addition, SDB in this cohort was mostly mild. It may be that fetal sequelae will only be unmasked in the setting of more severe degrees of SDB and/or underlying placental disease.

## Introduction

Sleep-disordered breathing (SDB) encompasses a spectrum of disorders characterised by increased upper airway resistance during sleep, and ranges from snoring to obstructive sleep apnoea (OSA). OSA occurs when the soft tissues of the upper airway collapse repeatedly during sleep, causing obstruction and cessation of airflow, often resulting in falls in blood oxygen saturation. SDB is being increasingly recognised as a potential risk factor in pregnancy, with reports of significant associations with gestational diabetes mellitus,[1] impaired fetal growth[2] and preterm birth.[3] Moreover physiological changes in pregnancy may increase the risk of SDB: these include weight gain, oedema, reduced upper airway dimensions,[4] and increased upper airway collapsibility due to hormonal changes.[5]

In the non-pregnant population, moderate-to-severe OSA is associated with hypertension and cardiovascular disease[6,7] due to a nocturnal cyclic pattern of hypoxia and hypercapnia, and recurrent surges of vasoconstriction. The degree of OSA appears to be linearly related to cardiovascular risk.[8] Accumulating evidence suggests this relationship may also exist in pregnancy with several studies reporting an association between SDB and gestational hypertensive disorders,[3,9,10] although obesity can act as a confounder to this relationship.[11] Hypertensive disorders of pregnancy (HDP), particularly preeclampsia, are associated with placental dysfunction resulting in impaired fetal growth and increased risks of acute compromise.[12–15] Whether co-existing SDB may increase these risks is currently unknown.

SDB may plausibly impact on fetal health in two ways. Firstly, recurrent episodes of hypoxaemia and cortical arousal lead to sympathetic activation and inflammation, leading to endothelial dysfunction.[16] The resultant placental dysfunction may lead to impaired fetal growth.[2] Secondly, such fetuses may be particularly sensitive to maternal hypoxaemia, which may lead directly to fetal hypoxia by reducing the availability of oxygen in the intervillous space. If sufficiently severe, this may result in fetal acidosis and asphyxial injury with potential long-term neurodevelopmental impairment and stillbirth.[17–19]

Few studies have investigated the impact of objectively-measured SDB on the health of the fetus, and results to date have been conflicting.[2,20–25] This may relate, in part, to challenges adjusting for comorbidities such as increasing BMI, which may mask FGR among women with SDB. For example, infants deemed appropriate for gestational age by standard growth curves may in fact be growth restricted after customisation for maternal obesity.[26,27] No studies to date have investigated the effect of SDB on the wellbeing of the fetus among mothers diagnosed with HDP. This is important because these fetuses might be expected to be more vulnerable to the sympathetic activation, endothelial dysfunction and hypoxemia that accompanies SDB. Given that HDP is a leading contributor to iatrogenic preterm birth, it would be helpful if we could identify reversible co-morbidities such as SDB that might enable safer prolongation of gestation. In this study, we proposed to determine whether the presence of objectively-confirmed SDB compromised the growth and well-being of the fetus among women with, and without, HDP.

## Materials and methods

This data is from a single-centre prospective matched case-control study conducted between October 2012 and October 2015, comparing women with the diagnosis of HDP to healthy women with uncomplicated pregnancies. The Human Research Ethics Committees at Austin Health, Mercy Hospital for Women and University of Melbourne approved the study and written informed consent was obtained from all participants.

Cases included women with a singleton pregnancy diagnosed with gestational hypertension (GH) or preeclampsia (PE) not requiring immediate delivery between 26 and 37 weeks’ gestation, and were recruited from the Pregnancy Day Assessment Centre or were inpatients at Mercy Hospital for Women, Melbourne, Australia. Hypertension in pregnancy was defined as systolic BP greater than or equal to 140mmHg and/or diastolic BP greater than or equal to 90mmHg, confirmed by a minimum of two readings over several hours. GH was defined as the new onset of hypertension after 20 weeks of gestation.[28] We used the most recent International Society for the Study of Hypertension in Pregnancy (ISSHP) definition of PE: new hypertension after 20 weeks gestation and one or more of the following new-onset conditions: i) proteinuria, ii) other maternal organ dysfunction (renal insufficiency, liver involvement or neurological complications), and iii) uteroplacental dysfunction–fetal growth restriction.[28] Women with chronic hypertension who developed superimposed PE were also eligible to participate as a PE case.

Control participants with normotensive uncomplicated pregnancies were one-to-one matched by BMI to each of the cases (within ±4 kg/m^2^, measured at the first antenatal appointment), and recruited from the antenatal outpatient clinic. Exclusion criteria included <18 years of age, multiple gestation, fetal abnormality or other maternal/fetal condition likely to mandate early or imminent delivery, and previous diagnosis of a sleep disorder.

GH and PE participants underwent overnight full polysomnography (PSG, ‘sleep study’) at their earliest convenience, to establish the presence or absence of SDB. Control women underwent PSG within ±4 weeks of gestational age to their matched hypertensive case participant. Attended overnight PSG was conducted in the Austin Health sleep laboratory using the Compumedics E series (Abbotsford, Victoria, Australia), or if preferred, unattended in the participant’s home with the Somté (Compumedics) portable sleep-monitoring device. Portable sleep monitoring systems are commonly used in clinical settings, and have been shown to have a high level of agreement with standard laboratory-based systems.[29,30] Inpatients were studied using the portable device. As per the American Academy of Sleep Medicine (AASM) criteria,[31] respiratory events were categorised as apnoeas (a decrease in airflow of ≥90% from baseline for ≥10 sec); hypopnoeas (decrease in airflow ≥30% from baseline for ≥10 sec and followed by either an oxygen desaturation of ≥3% or an EEG cortical arousal); and respiratory event related arousals (RERAs; a sequence of breaths lasting ≥10 sec characterised by increasing respiratory effort leading to an arousal from sleep). The number of apnoeas and/or hypopnoeas and/or RERAs per hour of sleep was calculated as the respiratory disturbance index (RDI). SDB was defined as an RDI of ≥5 events per hour, with secondary analyses performed with more significant SDB defined as RDI ≥15. SDB severity is classified as mild (RDI 5–14.9/hr), moderate (RDI 15–29.9/hr) and severe (RDI ≥30) in sleep literature.[32] The oxygen desaturation index (ODI) ≥3% was defined as the number of arterial oxygen desaturations of ≥3% from baseline, per hour of sleep.[31]

Time-synchronised cardiotocography (CTG) was performed using the Monica AN24 fetal heart rate (FHR) monitor (Monica Healthcare Ltd.) during the PSG to enable correlation of any FHR abnormalities with respiratory events and/or oxygen desaturations. All FHR traces were reviewed the following morning to ensure no abnormalities were present that mandated further management. CTG success was calculated as ‘minutes of FHR trace during sleep/minutes of sleep*100’ to give a value between 0 and 100%. The criteria for CTGs to be included in analysis was i) an overall CTG success rate of >50%, and ii) at least 3 x 1-hour blocks with >50% asleep and >80% successful FHR trace. This inclusion criterion was chosen as the FHR was difficult to interpret where there were large sections of missing data, and potential for FHR events to be missed.

*A priori* criteria for an abnormal FHR event potentially signifying fetal distress were as follows: Event 1 –prolonged bradycardia (defined as >15 bpm below baseline for ≥90 seconds and <5 minutes), Event 2 –recurrent severe variable decelerations (defined as a fall of >60 bpm from previous baseline and of >60 seconds duration, *and* at least 2 per 2 hours), Event 3 –repeated unprovoked or late decelerations accompanied by tachycardia or loss of variability, and Event 4 –decelerations lasting between 60–90 seconds and dropping below the baseline by >15 bpm.[33]

Fetal growth was monitored with ultrasound in the third trimester soon after recruitment, to enable sufficient time between third trimester growth assessment and delivery to identify slowing of fetal growth trajectory. The ultrasound estimate of fetal weight along with birthweight following delivery was customised for maternal height, pre-pregnancy (or if unknown, early pregnancy) weight, ethnicity, parity and fetal sex using the Australian dataset of the GROW software (www.gestation.net).[34]

*Fetal growth restriction* (FGR) was defined as a customised birthweight <10th centile for gestational age. Evidence of *slowed third trimester growth* was defined as a fall in customised centile of greater than a third (>33% decrease) between the third trimester ultrasound and birth. *Slowed third trimester growth* was calculated only for those participants where at least four weeks had lapsed between the third trimester ultrasound and birth. To facilitate standardised comparison, a rate of change in customised centile per day was calculated for each participant, and then multiplied by 42 to generate the estimated change in customised centile that would occur over exactly six weeks. This was chosen based on our previous studies examining maternal SDB and its impact on fetal growth,[2] and the importance of standardising assessment of fetal growth velocity.[35] Medical records were reviewed after birth to collect relevant delivery and perinatal outcomes.

Venous cord blood was collected at delivery and analysed for the fetal growth regulators IGF-1, IGF-2, IGFBP-1 and IGFBP-2. Free IGF-1 and IGF-2 determinations were performed using MILLIPLEX MAP Human IGF-I, II Magnetic Bead Panel kit (Millipore, Billerica, MA, USA). To separate IGFs from their binding proteins, all plasma samples underwent an acid-ethanol extraction procedure before measurement according to the manufacturer's instructions. All assays were read using the Bio-Plex workstation (Bio-Rad Laboratories, Hercules, CA, USA) and results analysed with Bio-Plex Manager (version 4.1.1) software. Intraassay and interassay coefficients of variation were less than 10%.

### Statistical analysis

All statistical analyses were performed with SPSS 21.0 (SPSS Inc., Chicago, Illinois). Values are given in means with standard deviations (*M* ± *SD)* or median and interquartile range (*Mdn (IQR)*) for non-normally distributed variables. A two-sided p value of less than 0.05 was considered to indicate statistical significance. Comparisons between groups on fetal outcomes were done using Fisher’s exact test of independence for categorical variables, independent-samples t-tests for normally-distributed continuous variables and Mann-Whitney U tests for non-normally distributed continuous variables. Extreme outlying values (z scores > 3.3) on cord blood data were excluded from analysis. Analysis of covariance (ANCOVA) was performed on the cord blood data for comparison between the HDP and normotensive group to control for differences in gestational age at sampling.

Due to a highly positively skewed distribution, but to preserve the breadth of the data, the rate of FHR events per hour of sleep was divided into three categories– 1) 0 events, 2) >0 to 0.5 events per hour, and 3) >0.5 events per hour. Ordinal logistic regression was used to assess the univariate relationships between the total FHR events per hour as an ordinal variable and SDB and demographic variables. Continuous RDI and ODI variables were transformed due to extreme skewness. Stepwise ordinal regression modelling was then performed with FHR events as the dependent variable, and explanatory variables with an α of less than 0.20 on univariate analysis were included.

## Results

**Fig 1:**
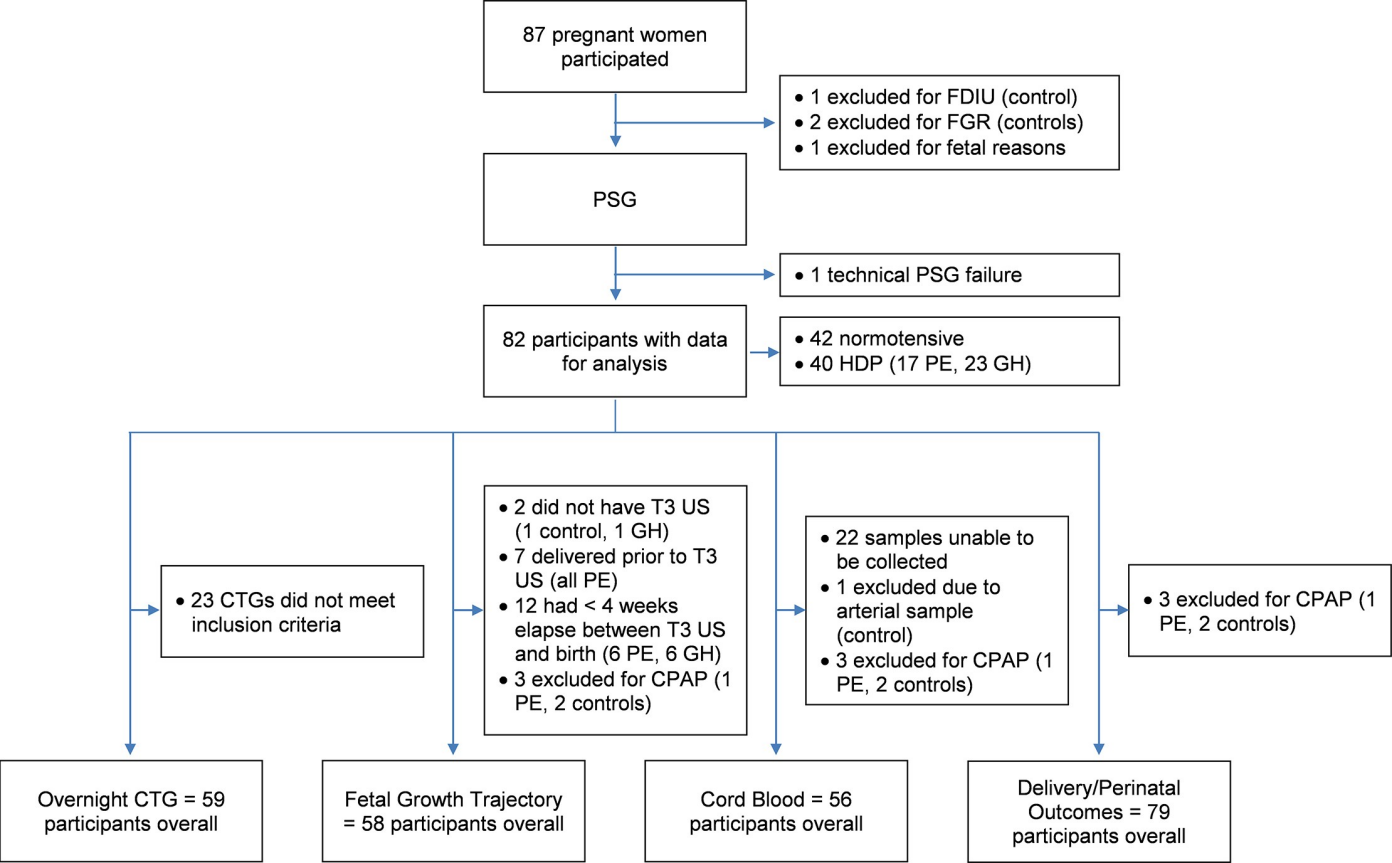
Flow chart of participant data available for each fetal outcome. FDIU = fetal death in utero, FGR = fetal growth restriction, GH = gestational hypertension, PSG = polysomnography, CTG = cardiotocography, T3 = third trimester, US = ultrasound, PE = preeclampsia, CPAP = continuous positive airway pressure.

A total of 87 pregnant women participated; a flowchart illustrating the data available at each step of analysis is shown in Fig 1.

Due to the case-control matched study design, there were no differences in age, parity, gestational diabetes, BMI and gestation at sleep study for the HDP versus normotensive control group (Table 1). SDB was diagnosed in 52.5% of the HDP group compared to 38.1% of the control group (p = .19) and there was no difference in RDI between the groups (Table 1). Once allocated into groups based on SDB, all demographic variables remained well-matched except for BMI at the time of the sleep study which was higher amongst those with SDB compared to No SDB in the HDP group. At the time of the sleep study, 23 of the hypertensive participants were taking anti-hypertensive medication. After undergoing PSG, three participants diagnosed with severe SDB commenced continuous positive airway pressure (CPAP) treatment and were subsequently excluded from all analyses apart from CTG analysis. As shown in Fig 1, perinatal outcomes were available on all other participants, but only 58 (73%) had greater than four weeks between growth scan and delivery enabling assessment of growth trajectory. Fifty-nine (72%) had satisfactory CTG recording which met our inclusion criteria and 56 (71%) provided a cord blood sample.

**Table 1:** Demographics for hypertensive disorders of pregnancy cases and normotensive controls stratified by SDB status.

		HDP				Controls		
	All HDP* (n = 40)	SDB (n = 21)	No SDB (n = 19)	p	All Controls (n = 42)	SDB (n = 16)	No SDB (n = 26)	p
Age (years)	32.7 ± 4.5	33.3 ± 4.7	32.0 ± 4.3	.38	33.1 ± 4.4	33.5 ± 3.9	32.9 ± 4.8	.67
Nulliparous	26 (65.0%)	14 (66.7%)	12 (63.2%)	1.0	20 (47.6%)	9 (56.3%)	11 (42.3%)	.53
GDM	8 (20.0%)	5 (23.8%)	3 (15.8%)	.70	9 (21.4%)	4 (25.0%)	5 (19.2%)	.71
BMI first appt	32.3 ± 7.3	33.8 ± 7.0	30.7 ± 7.4	.18	32.6 ± 7.0	34.1 ± 7.8	31.8 ± 6.4	.31
BMI at PSG	36.0 ± 7.0	38.1 ± 6.4	33.8 ± 7.0	.047	36.3 ± 6.1	37.9 ± 6.8	35.3 ± 5.5	.18
Gestation at PSG (weeks)	33.6 ± 3.5	34.0 ± 3.4	33.1 ± 3.6	.41	33.0 ± 2.4	33.2 ± 2.3	32.9 ± 2.6	.72
RDI/hr	5.2 (2.0, 14.8)	14.3 (7.5, 37.7)	1.9 (1.1, 3.1)	< .001	4.1 (2.2, 7.8)	9.2 (7.5, 28.8)	2.9 (1.3, 4.0)	< .001
ODI≥3% overall	2.1 (0.4, 10.1)	6.0 (2.1, 36.7)	1.0 (0.0, 1.8)	< .001	1.5 (0.6, 4.7)	8.7 (1.1, 29.1)	1.0 (0.2, 2.5)	.004
PE diagnosis	17 (42.5%)	7 (33.3%)	10 (52.6%)	.34	-	-	-	-

Values given as M ± SD, Mdn (IQR), or n (%). p value in table refers to SDB vs. No SDB groups

*No significant differences between HDP vs Controls

BMI measured at the first antenatal appointment was taken at a mean of 15.0 ± 2.6 weeks gestation. SDB = sleep-disordered breathing, HDP = hypertensive disorders of pregnancy, GDM = gestational diabetes mellitus, BMI = body mass index kg/m^2^, PSG = polysomnography, RDI = respiratory disturbance index, ODI = oxygen desaturation index.

### The impact of SDB on acute measures of fetal well-being: Cardiotocography

Twenty (33.9%) of the CTGs showed one or more of the four events indicating a potential episode of fetal compromise during sleep, but these were confined to Events 1 (prolonged bradycardia) and 4 (deceleration lasting between 60–90 sec with >15 bpm fall from baseline). The number of overall CTG events overnight per participant ranged from 0–5. As detailed in Table 2, there was no impact of HDP on the number of participants displaying any type of FHR event, or the total number of events per night (p = .78 and p = .19 respectively). Further, there was no impact of SDB, expressed as a dichotomous variable, on the number of HDP or normotensive participants displaying fetal heart rate events, nor the total number of events overnight.

**Table 2:** Cardiotocography variables for hypertensive disorders of pregnancy cases and normotensive controls stratified by SDB.

		HDP				Controls		
	All HDP* (n = 27)	SDB (n = 17)	No SDB (n = 10)	p	All Controls (n = 32)	SDB (n = 12)	No SDB (n = 20)	p
% CTG success	94.2 ± 9.0	94.3 ± 8.0	94.1 ± 11.0	.95	91.7 ± 10.8	88.9 ± 13.9	93.8 ± 8.2	.22
Hours of CTG	5.9 ± 1.3	5.7 ± 1.6	6.3 ± 0.8	.25	5.9 ± 1.2	5.8 ± 1.3	6.1 ± 1.1	.50
Any event								
n (%)	10 (37.0%)	6 (35.3%)	4 (40.0%)	1.0	10 (31.3%)	5 (41.7%)	5 (25.0%)	.44
Total events/night^#^	2.7 ± 1.4	2.7 ± 1.0	2.8 ± 2.1	.94	2.0 ± 0.8	2.0 ± 1.0	2.0 ± 0.7	1.0
Event 1								
n (%)	2 (7.4%)	1 (5.9%)	1 (10.0%)	1.0	1 (3.1%)	0 (0.0%)	1 (5.0%)	1.0
Event 2								
n (%)	0 (0.0%)	0 (0.0%)	0 (0.0%)	-	0 (0.0%)	0 (0.0%)	0 (0.0%)	-
Event 3								
n (%)	0 (0.0%)	0 (0.0%)	0 (0.0%)	-	0 (0.0%)	0 (0.0%)	0 (0.0%)	-
Event 4								
n (%)	10 (37.0%)	6 (35.3%)	4 (40.0%)	1.0	10 (31.3%)	5 (41.7%)	5 (25.0%)	.44

Values given as M ± SD, Mdn (IQR) or n (%). p value in table refers to SDB vs. No SDB groups

SDB = sleep-disordered breathing, HDP = hypertensive disorders of pregnancy, CTG = cardiotocography, BMI = body mass index kg/m^2^, RDI = respiratory disturbance index.

*No significant differences between HDP vs Controls

^#^only those who had fetal heart rate events.

Significant univariate predictors of FHR decelerations overnight were nulliparity, earlier gestational age, diagnosis of PE and FGR at birth, but not presence or severity of SDB in terms of RDI or ODI3 (S1 Table). A stepwise selection model confirmed FGR at birth as the strongest predictor of number of FHR events per hour on CTG (χ^2^ = 18.63, p = .001, R^2^ = 0.33; Table 3), with the diagnosis of PE no longer a significant factor. Over 60% of mothers with a growth-restricted infant displayed evidence of CTG abnormalities during sleep, versus only 26% of well-grown fetuses (p = .04). SDB did not exacerbate FHR events in FGR fetuses (number of events—SDB = 2.7 ± 1.5 vs. No SDB = 2.8 ± 1.6, p = .23) overall, although severity of SDB became a significant predictor of FHR deceleration in the multivariate model (Table 3). This relationship was limited to the control group (r = .44, p = .02, n = 28); examination of the linear relationship revealed that only normotensive women with an RDI ≥3.5 were observed to have adverse nocturnal FHR events.

**Table 3:** Factors associated with number of fetal heart rate events per hour on cardiotocography on stepwise ordinal regression modelling.

Variable	*B*	Wald χ^2^ test	aOR (*95% CI*)	p
Gestation at CTG*	-0.22	2.94	0.57 (0.30–1.08)	.09
Nulliparous	1.51	5.02	4.53 (1.21–17.01)	.03
FGR at birth	1.67	5.20	5.31 (1.26–22.26)	.02
RDI log*^#^	0.62	4.51	2.12 (1.06–4.24)	.03
Threshold coefficient = 0	-5.77	1.77		
Threshold coefficient = 1	-4.22	0.96		

N = 59. aOR = adjusted odds ratio, CTG = cardiotocography, FGR = fetal growth restriction, RDI = respiratory disturbance index.

*OR for continuous variables indicate the change in odds for an increase of one standard deviation.

^#^logarithmic transformation due to extreme skewness

Threshold coefficient = 0 –the odds of FHR events per hour being 0.

Threshold coefficient = 1 –the odds of FHR events per hour being 0.5 per hour or less.

### The impact of SDB on fetal growth and perinatal outcomes

As expected, women with HDP had infants with a lower birthweight, a higher incidence of FGR at birth and evidence of lower customised birthweight centiles and slowed fetal growth between the third trimester ultrasound and birth, compared to normotensive controls (Table 4). However, the co-existence of SDB with HDP did not appear to impact further on birthweight or fetal growth. In fact, among the women with HDP, co-existing SDB was associated with significantly better perinatal outcomes: larger customised birthweight centiles and fewer FGR at birth than those without SDB. This association persisted even after adjustment for the higher proportion of PE participants in the No SDB group (p = .052). This observation was not seen among control participants where there were no significant differences in birthweight centile or fetal growth between the SDB and No SDB groups (Table 4).

**Table 4:** Measures of fetal growth and perinatal outcomes for those with and without SDB in the hypertensive disorders of pregnancy and control groups.

		HDP				Controls		
	All HDP (n = 39*)	SDB (n = 20)	No SDB (n = 19)	p	All Controls (n = 40*)	SDB (n = 14)	No SDB (n = 26)	p
*Fetal Growth*								
Birth Gestation (weeks)^a^	35.6 ± 3.9	36.3 ± 4.0	34.9 ± 3.8	.26	39.5 ± 1.2	39.4 ± 1.3	39.6 ± 1.1	.69
Birthweight (g)^a^	2561.2 ± 1141.6	2881.6 ± 1188.6	2223.9 ± 1012.9	.07	3514.4 ± 493.1	3450.2 ± 485.3	3549.0 ± 503.2	.55
Birthweight cust. centile (%)^d^	30.0 ± 35.6	43.2 ± 38.3	16.2 ± 27.0	.015	43.5 ± 27.3	41.9 ± 29.0	44.3 ± 26.9	.79
FGR at recruitment^a^	10 (25.6%)	4 (20.0%)	6 (31.6%)	.48	0 (0.0%)	0 (0%)	0 (0%)	1.0
FGR at birth (<10th cust. centile)^b^	19 (48.7%)	6 (30.0%)	13 (68.4%)	.026	6 (15.0%)	2 (14.3%)	4 (15.4%)	1.0
Impaired fetal growth (FGR or fall in cust. centile >33%)^a^	25 (66.7%)	10 (50.0%)	15 (78.9%)	.18	9 (22.5%)	4 (28.6%)	5 (19.2%)	.69
Participants with T3 scan**		*n = 12*	*n = 7*			*n = 14*	*n = 25*	
T3 scan gestation (weeks)	32.2 ± 2.6	32.6 ± 2.7	31.6 ± 2.5	.44	32.5 ± 2.1	32.5 ± 1.4	32.5 ± 2.4	.99
T3 cust. centile (%)	59.7 ± 32.1	66.3 ± 29.6	48.3 ± 35.5	.25	53.6 ± 24.2	49.0 ± 26.8	56.2 ± 22.7	.38
Birthweight cust. centile (%)	47.7 ± 35.9	54.4 ± 35.2	36.2 ± 36.8	.30	43.2 ± 27.6	41.9 ± 29.0	44.0 ± 27.4	.83
Change in cust. centile per day	-0.33 ± 0.83	-0.36 ± 0.98	-0.28 ± 0.52	.84	-0.22 ± 0.42	-0.14 ± 0.44	-0.26 ± 0.41	.43
Fall in cust. centile > 33% over 6 weeks^d^	6 (31.6%)	4 (33.3%)	2 (28.6%)	1.0	4 (10.3%)	2 (14.3%)	2 (8.0%)	.61
*Perinatal Outcomes*								
Preterm Birth (< 37 weeks) ^a^	18 (46.2%)	6 (30.0%)	12 (63.2%)	.056	0 (0.0%)	0 (0%)	0 (0%)	-
Caesarean %^b^	30 (76.9%)	15 (75.0%)	15 (78.9%)	1.0	17 (42.5%)	6 (42.9%)	11 (42.3%)	1.0
Emergency Caesarean^b^	19 (48.7%)	9 (45.0%)	10 (52.6%)	.75	8 (20.0%)	3 (21.4%)	5 (19.2%)	1.0
Apgar 1 min ≤ 7	12 (30.8%)	8 (40.0%)	4 (21.1%)	.30	5 (12.8%)	3 (21.4%)	2 (8.0%)	.33
Apgar 5 min ≤ 7	3 (7.7%)	3 (15.0%)	0 (0%)	.23	1 (2.6%)	1 (7.1%)	0 (0%)	.36
NICU^b^	8 (20.5%)	3 (15.0%)	5 (26.3%)	.45	0 (0.0%)	0 (0%)	0 (0%)	-
SCN^c^	12 (30.8%)	5 (25.0%)	7 (36.8%)	.50	3 (7.7%)	3 (21.4%)	0 (0%)	.04

Values given as M ± SD or n (%). p value in table refers to SDB vs. No SDB groups. HDP = hypertensive disorders of pregnancy, SDB = sleep-disordered breathing, cust. = customised, FGR = fetal growth restriction, NICU = neonatal intensive care unit, SCN = special care nursery.

*excluding three participants commenced on CPAP (1 x HDP, 2 x controls)

** Only participants who had an ultrasound performed at least 4 weeks prior to delivery were included

^a^HDP vs Controls p < .001

^b^HDP vs Controls p < .01

^c^HDP vs Controls p < .05

^d^HDP vs Controls p = .06

Similarly, women with HDP had a significantly higher risk of preterm birth, caesarean delivery and SCN or NICU admission compared to normotensive controls (Table 4). Again, co-existing SDB did not amplify the risks for those with HDP, rather SDB was associated with a lower incidence of pre-term birth. Apart from a higher rate of SCN admission, there were no adverse perinatal outcomes for normotensive women with SDB.

Secondary analysis of participants with an RDI ≥15 showed no negative effects of moderate to severe SDB on fetal growth or perinatal outcomes, again showing evidence of larger customised birthweight centile and fewer FGR at birth, compared to mothers with an RDI <15 (S2 Table).

### The impact of SDB on biomarkers of fetal growth

**Fig 2:**
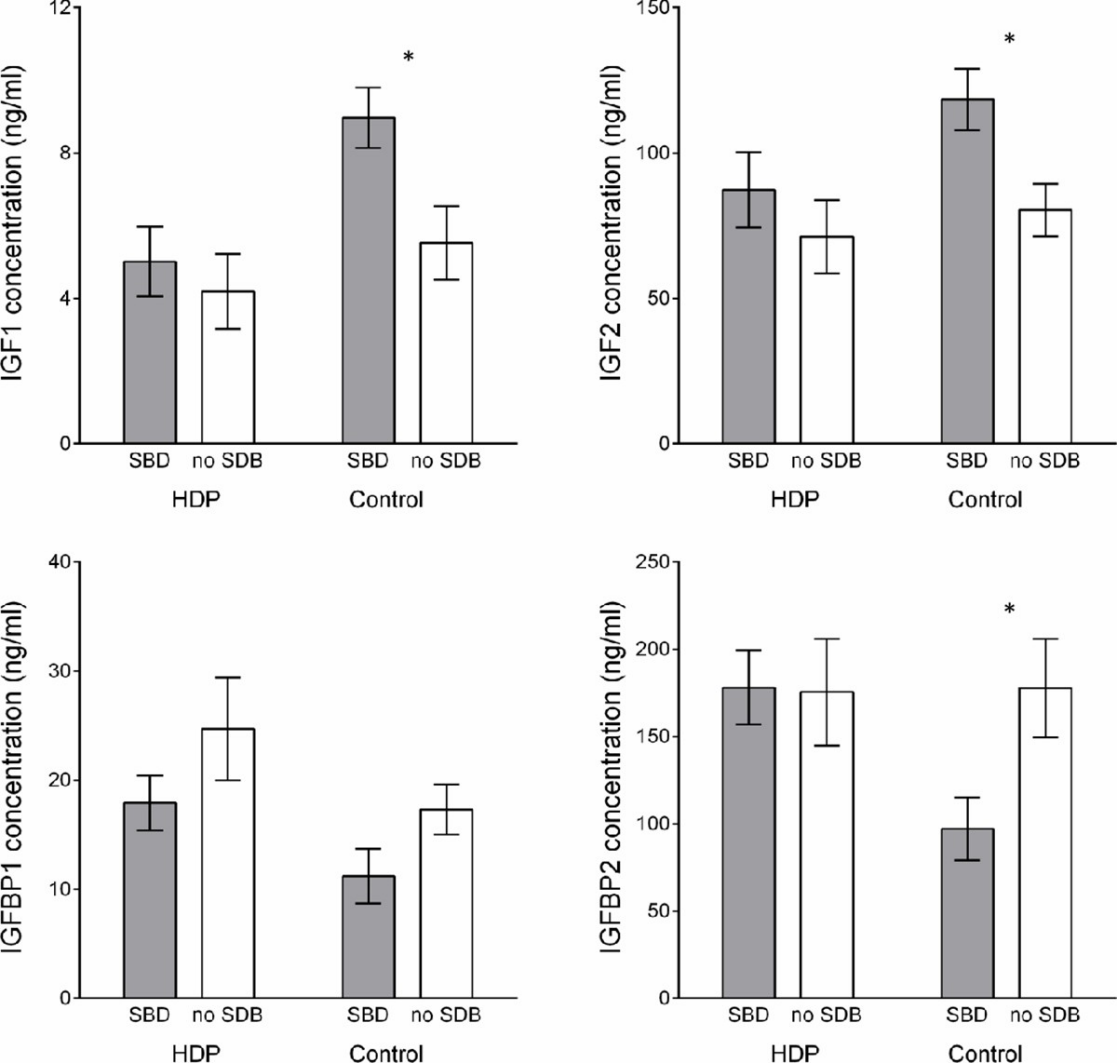
Mean ± SEM insulin-like growth factor 1 and 2 (IGF-1, IGF-2) and insulin-like growth factor binding protein 1 and 2 (IGFBP-1, IGFBP 2) for the SDB vs. No SDB group within the HDP and control group (*p value = .02). HDP group–SDB n = 17, No SDB n = 13. Control group SDB n = 8, No SDB n = 17, except for IGFBP-1 where HDP group–No SDB n = 12, Control group No SDB n = 16. SDB = sleep-disordered breathing, HDP = hypertensive disorders of pregnancy.

After adjusting for gestational age, there was no difference in markers of fetal growth between HDP and normotensive women (Table 5). The gestational age at which the cord blood sample was taken did not differ by SDB status. As shown in Fig 2, amongst those with HDP there was no impact of SDB on the IGF axis. However, in the control group, the participants with SDB had significantly higher IGF-1, IGF-2 and lower IGFBP-2.

**Table 5:** Biomarkers of fetal growth in cord blood across hypertensive groups.

	HDP (n = 30)	Normotensive (n = 25)	p
IGF-1 (ng/ml)	4.7 ± 3.8	6.6 ± 4.0	.07^†^
IGF-2 (ng/ml)	80.4 ± 49.9	92.6 ± 39.0	.32
IGFBP-1 (ng/ml)	20.7 ± 13.3	15.2 ± 8.9	.09^‡^
IGFBP-2 (ng/ml)	177.0 ± 96.2	152.0 ± 105.9	.37
Gestation Sample Taken (weeks)	36.0 ± 3.9	39.2 ± 1.1	< .001

Values given as M ± SD, Mdn (IQR), or n (%). HDP = hypertensive disorders of pregnancy

IGF = insulin-like growth factor, IGFBP = insulin-like growth factor binding protein.

^†^ after ANCOVA with covariate of gestational age at sample, p = .85.

^‡^ after ANCOVA with covariate of gestational age at sample, p = .41.

## Discussion

This is one of the few prospective studies that has combined comprehensive assessment of fetal growth and wellbeing with objectively-measured SDB, and the first study thus far to have examined this relationship in women with HDP. We found that women with HDP were more likely to have smaller infants with a higher incidence of FGR, however the presence of co-existing SDB did not adversely impact on fetal growth or size at birth. Similarly, fetuses known to have FGR were more likely to display nocturnal FHR decelerations but this was not exacerbated by the presence of SDB. Furthermore, no adverse impact on perinatal outcomes was seen in normotensive women with SDB.

FGR at birth was the strongest risk factor for FHR decelerations at night. Given that FGR is associated with severe underlying placental dysfunction, and strongly associated with adverse perinatal outcome including stillbirth,[36,37] it might be expected that FGR fetuses have the least oxygen reserve to withstand further physiological insults at night. This also underscores why night time may be a particularly risky time for FGR fetuses—the importance of maternal sleep has been highlighted by Warland et al.[38] finding that more than half of mothers experiencing stillbirth believed that their baby died at night. For this important reason, we had a particular interest in seeing whether maternal SDB would be more poorly tolerated by a fetus already suffering uteroplacental insufficiency, as demonstrated previously by Fung et al.[2] In our study however, FGR fetuses were not further adversely impacted by maternal SDB. This may be because the more severely growth-restricted fetuses were most difficult to obtain a successful CTG trace, and SDB in this cohort was generally mild. Nevertheless, this association between customised centile of <10% at birth and fetal heart rate events in late pregnancy provides an avenue to explore with regard to nocturnal FHR assessment as an early predictor of FGR risk at birth.

SDB severity, expressed as a continuous measure, was independently associated with nocturnal FHR events, mainly in normotensive women. Despite this, it should be kept in mind that the FHR events observed were mild, with the two most ‘severe’ types of FHR decelerations not observed at all (recurrent severe variable decelerations and repeated unprovoked decelerations or late decelerations). It is likely that the appropriately-grown fetus is well protected from transient maternal hypoxaemia, due to a combination of fetal adaptive behaviours such as increasing heart rate and cardiac output to increase O_2_ uptake from its placenta,[39] and the high affinity fetal haemoglobin-oxygen dissociation curve.[40] Olivarez et al.[25] demonstrated that FHR events among 20 women diagnosed with SDB were variable decelerations appropriate for gestational age and therefore not pathologic. Two early case studies[41,42] demonstrating substantial FHR decelerations in association with maternal sleep apnoea presented women with very severe SDB–yet the seven women with the most severe disease (RDI>30) in our study still only demonstrated minor fetal events.

The fact that fetal outcomes were not worse in HDP associated with SDB is unexpected. The absence of any relationship may reflect relatively mild SDB disease; however the lack of association between SDB and poor fetal health remained even across the more severe disease subtype. While we may have been underpowered for many of the individual adverse outcomes, there were no apparent differences across fetal outcomes including fetal growth restriction, preterm birth, mode of delivery and NICU admission, when taken collectively.

Previous studies by Fung et al.[2] and Kneitel et al.[43] found that SDB was associated with slowed fetal growth across pregnancy rather than low birthweight. While we found SDB had no pathological effects on fetal growth trajectory, this may have been due to the population targeted and study design. A limitation of monitoring third trimester growth in HDP, particularly PE, is that the maternal and fetal condition often mandates very pre-term delivery. Hence, the third trimester growth trajectory analysis only included fetuses well enough to complete a third trimester ultrasound and remain in utero for at least another two weeks before delivery. Consequently a number of FGR fetuses, particularly within the No SDB group, were excluded.

One interesting result from this study was that hypertensive women with at least mild SDB had larger newborns even after fetal weight was customised for maternal characteristics such as obesity, and they were less likely to be premature. While this may be a chance finding, it is intriguing to consider whether the presence of SDB may confer a better prognosis in HDP. An association between SDB and increased birthweight has been demonstrated recently in women with mild OSA,[44] with a potential explanation relating to an association between increased placental weight and maternal OSA severity, possibly mediated by placental leptin overexpression.[45] Despite no increase in birthweight, we also demonstrated heightened levels of important fetal growth regulators IGF-1 and IGF-2 with a corresponding decrease in IGFBP-2 in normotensive women with SDB. Among women with preeclampsia, the intermittent hypoxic challenge of SDB may result in a less severe placental phenotype than the chronic placental dysfunction and hypoxia/ischaemia model classically associated with PE. HDP linked to SDB may have a more benign course with placental and maternal endothelial re-oxygenation daily, whereas PE resultant from early uteroplacental insufficiency would be chronically hypoxic. While we acknowledge this interpretation is speculative, it would be amenable to further investigation in appropriately designed laboratory studies.

The contrasting findings in the hypertensive and normotensive mothers on the impact of SDB on fetal growth measures contribute to the current uncertainties reported in the literature. Studies have reported varying results: ranging from a link between diagnosed maternal SDB and small for gestational age infants[21,46] markers of placental tissue hypoxia[47] and lower levels of estriol, a marker of fetoplacental unit wellbeing,[48] through to no relationship between SDB and fetal growth.[20,23,24,49] These inconsistencies may relate to inadequate adjustment for confounding variables, the predominance of mild SDB or differing methodologies, however it is clear the answer remains elusive and further study is warranted.

The lack of association between SDB and adverse perinatal outcomes in our study helps inform the debate regarding treatment recommendations. Even if it were feasible to screen and treat all pregnant women affected by SDB, adherence to CPAP may be poor and any benefit to the fetus may only be modest. In our study, of the 15 women with moderate to severe SDB who attended follow-up, only three women accepted CPAP treatment. This problem has been identified in other studies[23] and suggests that stronger links between SDB and poor pregnancy outcomes need to be demonstrated before pregnant women regard treatment as an acceptably high priority.

### Strengths and limitations

This is the first study to examine how SDB may affect fetal growth and perinatal outcomes among women with hypertension in pregnancy. We employed comprehensive assessments to determine the role SDB plays on acute and chronic measures of placental insufficiency. In particular, our study used a strict definition for acceptable and interpretable FHR tracing and we successfully gathered multiple hours of continuous nocturnal CTG monitoring compared to previous attempts.[25,50] We based our comprehensive technique for investigating whether maternal SDB affects fetal growth on Fung et al,[2] with the inclusion of fetal growth trajectory as an indicator of placental insufficiency, and collection of cord blood to analyse hormonal markers of fetal growth. In pregnancies with appropriate for gestational age sized fetuses, slowing third trimester growth has been associated with adaptive cerebral blood flow patterns in the fetus associated with in utero hypoxia, increased rates of fetal compromise during labour,[51] neonatal acidosis and reduced neonatal body fat percentage.[35] Another important consideration for our study was to customise both the ultrasound estimate of fetal weight and birthweight according to maternal characteristics known to affect birthweight. This is particularly important in larger women (a high proportion of our sample) where significant growth restriction may be present, yet the fetus is not below the 10th percentile at delivery based on population standards.

Importantly, any short or long-term effect of maternal hypoxaemia may have been attenuated by the relatively mild degree of SDB in this study. The average level of nocturnal hypoxaemia experienced was quite minimal, with a median of only three episodes of oxygen desaturations per hour. In comparison, mechanistic animal models investigating the impact of hypoxia on the fetus typically induce very severe conditions[52,53] unlike that seen in human pregnancy, where the interaction between maternal contributors and underlying placental health is likely much more subtle.

Sample size was a notable shortcoming of this study. Overnight PSG is considered the gold standard method of diagnosing SDB but is intensive. The amount of data available for each outcome was limited by a number of factors; as illustrated in Fig 1. Despite these limitations, we were able to comprehensively interrogate the impact of coexisting SDB on perinatal outcomes with multiple outputs and thus these findings add useful data to the existing literature, and challenge existing assumptions about the influence of SDB as an independent comorbidity in high risk pregnancies.

### Conclusions

Using comprehensive assessments of acute and chronic measures of placental insufficiency and fetal well-being, we have not found that the presence of SDB as an independent comorbidity has a detrimental effect on perinatal health, including among hypertensive pregnancies. We found no evidence to support a relationship between SDB and CTG abnormalities, fetal growth trajectory and growth restriction at birth, or perinatal outcomes. The fetus has considerable adaptive capacity to withstand in utero hypoxia, which may explain our mostly negative findings. In our study, FGR was uncommon and mostly mild, at the time of PSG. SDB was also mostly mild. It seems likely that fetal sequelae will only be unmasked in the setting of either more severe degrees of SDB and/or severe underlying FGR and placental disease. Such studies are already underway and will help inform the place, and value, of screening and treating SDB in high risk pregnancies.

## Supporting information

S1 Table Univariate relationships between predictor variables and number of fetal heart rate events per hour on cardiotocography. (DOCX)

S2 Table Key fetal outcomes for those with and without maternal SDB at an RDI ≥ 15. (DOCX)
