# Supplementary material for: The presence of coexisting sleep-disordered breathing among women with hypertensive disorders of pregnancy does not worsen perinatal outcome
Source: PLoS One. 2020 Feb 26;15(2):e0229568. doi: 10.1371/journal.pone.0229568 (PMC7043804; doi:10.1371/journal.pone.0229568)
Supplement: S1 Table — (DOCX) [file pone.0229568.s001.docx]

S1 Table. Univariate Relationships Between Predictor Variables and Number of Fetal Heart Rate Events per Hour on Cardiotocography

| Variable | Units or Category (*Range/SD*) | OR (*95% CI*) | p |
| --- | --- | --- | --- |
| Age* | yr (21 - 40/4.4) | 1.04 (0.61 – 1.78) | .89 |
| Nulliparous | Yes or No | 4.79 (1.38-16.61) | .014 |
| BMI at CTG* | kg/m^2^ (22.7 – 53.1/6.2) | 0.72 (0.41–1.28) | .27 |
| Gestation at CTG* | weeks (26.7 – 37.7/2.6) | 0.56 (0.33–0.96) | .036 |
| Antihypertensives | Yes or No | 2.07 (0.62 – 6.95) | .24 |
| HDP |  |  |  |
| GH vs. control | Yes or No | 1.00 (0.30 – 3.33) | 1.0 |
| PE vs. control | Yes or No | 6.79 (1.37 – 33.75) | .019 |
| FGR at birth | Yes or No | 4.58 (1.36 – 15.46) | .014 |
| RDI ≥ 5 | Yes or No | 1.49 (0.52 – 4.31) | .46 |
| RDI log* | Index (-0.69 – 4.69/1.22) | 1.45 (0.85 – 2.47) | .17 |
| ODI ≥3% log* | Index (-4.61 – 4.67/2.51) | 1.26 (0.72 – 2.20) | .40 |

*Note.* N = 59. OR = odds ratio, BMI = body mass index, CTG = cardiotocography, HDP = hypertensive disorders of pregnancy, GH = gestational hypertension, PE = preeclampsia, FGR = fetal growth restriction, RDI = respiratory disturbance index, ODI = oxygen desaturation index.

*OR for continuous variables indicate the change in odds for an increase of one standard deviation.
