# Supplementary material for: The presence of coexisting sleep-disordered breathing among women with hypertensive disorders of pregnancy does not worsen perinatal outcome
Source: PLoS One. 2020 Feb 26;15(2):e0229568. doi: 10.1371/journal.pone.0229568 (PMC7043804; doi:10.1371/journal.pone.0229568)
Supplement: S2 Table — (DOCX) [file pone.0229568.s002.docx]

S2 Table. Key Fetal Outcomes for those with and without Maternal SDB at an RDI ≥ 15

|  | *SDB  (n = 12) | No SDB  (n = 67) | p |
| --- | --- | --- | --- |
| RDI/hr | 32.9 (23.2 – 52.6) | 3.7 (1.9 – 6.6) | <.001 |
| ODI ≥ 3% | 32.9 (21.9 – 42.2) | 1.3 (0.3 – 3.2) | <.001 |
| Birth gestation (weeks) | 38.0 ± 1.3 | 37.5 ± 3.7 | .43 |
| Birthweight (g) | 3313.8 ± 625.5 | 2995.5 ± 1041.7 | .31 |
| Birth cust. centile (%) | 52.6 ± 30.9 | 34.0 ± 31.8 | .06 |
| FGR at recruitment | 1 (8.3%) | 9 (13.4%) | 1.0 |
| FGR at birth (<10^th^ cust. centile) | 1 (8.3%) | 24 (35.8%) | .09 |
| Impaired fetal growth (FGR or fall in cust. centile >33%) | 2 (16.7%) | 32 (47.8%) | .06 |
| *Participants with valid T3 scan* | *n = 9* | *n = 49* |  |
| T3 cust. centile (%) | 54.7 ± 32.1 | 55.7 ± 26.2 | .92 |
| Birthweight cust. centile (%) | 47.4 ± 30.5 | 44.2 ± 30.6 | .77 |
| Change in cust. centile per day (%) | -0.23 ± 0.73 | -0.26 ± 0.56 | .91 |
| Fall in cust. centile >33% over 6 weeks | 1 (11.1%) | 9 (18.4%) | 1.0 |
| *Perinatal Outcomes* | *n = 12* | *n = 67* |  |
| Preterm Birth <37 weeks | 1 (8.3%) | 17 (25.4%) | .28 |
| Caesarean % | 8 (66.7%) | 39 (58.2%) | .75 |
| Emergency caesarean | 2 (16.7%) | 25 (37.3%) | .20 |
| Apgar 1 min ≤ 7 | 2 (16.7%) | 15 (22.7%) | 1.0 |
| Apgar 5 min ≤ 7 | 0 (0.0%) | 4 (6.1%) | 1.0 |
| NICU admit | 0 (0.0%) | 8 (12.1%) | .35 |
| SCN admit | 2 (16.7%) | 13 (19.7%) | 1.0 |
| *Cord Blood* | *n = 11* | *n = 44* |  |
| IGF-1 (pg/ml) | 6564.5 ± 3274.9 | 5303.7 ± 4137.3 | .35 |
| IGF-2 (pg/ml) | 108175.5 ± 41213.7 | 80394.1 ± 44939.5 | .07 |
| IGFBP-1 (ng/ml) | 12.5 ± 6.7 | 19.7 ± 12.3 | .07 |
| IGFBP-2 (ng/ml) | 159.1 ± 102.1 | 167.3 ± 101.3 | .81 |
| Gestation Sample Taken | 38.1 ± 1.3 | 37.3 ± 3.7 | .27 |

*Note.* Values given as M ± SD, Mdn (IQR), or n (%). SDB = sleep-disordered breathing, RDI = respiratory disturbance index, ODI = oxygen desaturation index, cust. = customised, FGR = fetal growth restriction, T3 = third trimester, NICU = neonatal intensive care unit, SCN = special care nursery, IGF = insulin-like growth factor, IGFBP = insulin-like growth factor binding protein.

*excludes 3 CPAP users.
